# Effects of anxiety, depression, social support, and physical health status on the health-related quality of life of pregnant women in post-pandemic Korea: a cross-sectional study

**DOI:** 10.4069/kjwhn.2023.09.11

**Published:** 2023-09-26

**Authors:** Hyun Kyoung Kim, Geum Hee Jeong, Hye Young Min

**Affiliations:** 1 College of Nursing, Kongju National University, Kongju, Korea; 2 School of Nursing and Research Institute in Nursing Science, Hallym University, Chuncheon, Korea; 3 College of Nursing, Ewha Woman’s University, Seoul, Korea

**Keywords:** Depression, Physical health, Pregnant women, Quality of life, Social support

## Abstract

**Purpose:**

This study aimed to examine the effects of anxiety, depression, social support, and physical health status on the health-related quality of life of Korean pregnant women using Spilker’s quality of life model.

**Methods:**

This was a cross-sectional study with a correlational design. The participants included 166 pregnant women who were recruited via convenience sampling at two healthcare centers in South Korea. Questionnaires were collected from April 22 to May 29, 2023, in two cities in South Korea. The EuroQol-5D-3L, General Anxiety Disorder-7, Patient Health Questionnaire-2, Perceived Social Support through Others Scale-8, and EuroQol visual analog scale were used to assess the study variables. The t-test, Pearson correlation coefficients, and multiple regression tests were conducted using IBM SPSS ver. 26.0.

**Results:**

Statistically significant correlations were identified between the health-related quality of life of pregnant women and anxiety (r=.29, *p*<.001), depression (r=.31, *p*<.001), social support (r=–.34, *p*<.001), and physical health status (r=–.44, *p*<.001). Physical health status (β=–.31, *p*<.001) and social support (β=–.21, *p*=.003) had the greatest effect on health-related quality of life (F=15.50, *p*<.001), with an explanatory power of 26.0%.

**Conclusion:**

The health-related quality of life of pregnant women was affected by social support and physical health status. This study demonstrated that physical health and social support promotion can improve the health-related quality of life of pregnant women. Healthcare providers should consider integrating physical health into social support interventions for pregnant women in the post-pandemic era.

## Introduction

Pregnant women tend to experience physical discomfort, including decreased mobility, due to the sharp increase in estrogen and progesterone and rapid weight gain. In addition to physical changes, they also experience developmental difficulties as they adjust psychologically to changes in the family structure and psychological difficulties due to ambivalence [1]. Since the coronavirus disease 2019 (COVID-19) pandemic, pregnant women tend to experience more anxiety and depression than other demographic groups due to deteriorated mental health resulting from social isolation and quarantine measures [2]. Therefore, they are a vulnerable population with a high risk of deteriorating mental health in the wake of the COVID-19 pandemic [3].

Pregnant women are at risk of a lower health-related quality of life due to potential physical and mental health problems that occur during pregnancy [4]. Health-related quality of life (HRQoL) can be conceptualized as an individual’s level of functioning and subjective perception of their overall well-being across multiple dimensions of health, including physical, mental, and social domains [5]. A meta-analysis of the HRQoL of pregnant women identified demographic factors such as age and gestational age, social factors such as family and friends, physical factors such as nausea and pain, and psychological factors such as anxiety and depression to be the factors that most affect HRQoL [4]. Another study showed that higher HRQoL was associated with the third trimester of pregnancy compared to the second trimester, maternal age of 26 to 30 years compared to other ages, and not having a job compared to having a job [6]. In a systematic review by Boutib et al. [7], the physical factors that affected the HRQoL of pregnant women included nausea, back pain, and pelvic pain; the demographic factors included advanced gestational age and multiple previous deliveries, and the psychological factors included anxiety, and depression. The factors that positively affected HRQoL were social support, physical exercise, and good sleep [7]. However, the influential factors are not consistent across countries, and few studies have simultaneously examined the physical, mental, and social factors related to pregnant women in Korea. Additionally, there is a lack of research on the HRQoL of pregnant women in the post-pandemic era.

**Figure 1.**
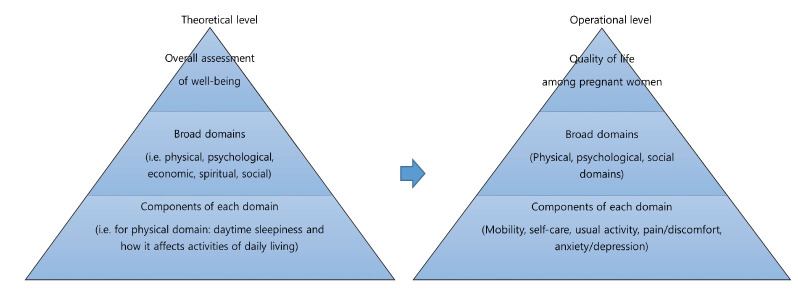
Conceptual framework according to Spilker’s Quality of life model (1996).

Therefore, this study aimed to examine the effects of perceived physical health status and psychological factors such as anxiety and depression on the HRQoL of pregnant women. Spilker’s [8] Quality of life (QoL) model was applied as the theoretical framework in this study. The QoL domains were organized in a pyramid model. At the bottom of the pyramid were the elements of each domain; in the middle were the broader domains of mental health, social health, and physical health; and at the top was overall well-being. In this study, we applied a conceptual framework using anxiety and depression to assess mental health, social support to assess social health, and physical health status to assess physical health as the factors that affect pregnant women’s HRQoL (Figure 1). This study aimed to identify the effects of psychological health, social support, and physical health status on the HRQoL of pregnant women. The determinants of HRQoL in pregnant women identified in this study will serve as a basis for the development of nursing interventions to improve their HRQoL in the domains of mental, social, and physical health.

This study aimed to explore the impact of anxiety, depression, social support, and physical health status on the quality of life of pregnant women in the post-pandemic era. The study’s specific objectives were as follows: (1) to measure the anxiety, depression, social support, physical health status, and HRQoL of pregnant women; (2) to analyze the relationships among anxiety, depression, social support, physical health status, and HRQoL in pregnant women; and (3) to identify the effects of anxiety, depression, social support, and physical health status on the HRQoL of pregnant women.

## Methods

This study was approved by the Institutional Review Board of Hallym University (HIRB-2023-020). Informed consent was obtained from the participants.

### Study design

This is a correlational study that used a cross-sectional survey to analyze the factors that affect the HRQoL of pregnant women. This study was conducted according to the STROBE (Strengthening the Reporting of Observational Studies in Epidemiology) reporting guidelines [9].

### Participants

The participants were selected via convenience sampling from the antenatal education programs at public health centers located in Chuncheon, Gangwon Province, and Gongju, Chungcheongnam Province in South Korea. The researcher visited the director of the public health center’s maternal and child center to outline the purpose of the study, the data collection period, and the research methods and obtained permission from the director to conduct the study. After explaining the purpose and methods of the study to the women who attended the antenatal education program at the public health center, the researcher asked if they would participate in the study and obtained their written consent. Trained research assistants shared a description of the study and a written consent form, allowing the mothers enough time to understand the study objectives and procedures, and distributed questionnaires to those who voluntarily agreed to participate. The inclusion criteria were (1) those aged 20 years or older, (2) those who agreed to the purpose of the study, and (3) pregnant women who could read and write Korean. The exclusion criteria were (1) those with health problems (gestational hypertension, preterm labor, miscarriage) in a previous pregnancy, (2) those experiencing maternal health problems during their current pregnancy, and (3) those experiencing fetal health problems during their current pregnancy. The scope of the participants’ health problems was based on the diseases that affect quality of life from a meta-analysis by Li et al. [10].

The number of participants was calculated using G*Power [11], with an effect size of 0.21, based on a range of effect sizes of 0.21 to 13.10 in a previous study on the impact of maternal health on quality of life [12], using regression analysis, α of 0.05, power of 0.95, and 13 variables (age, number of children, gestational age, trimester, number of pregnancies, number of deliveries, occupation, past history, present disease, depression, anxiety, social support, and physical health status), resulting in a total of 139 participants. An additional 20% was added to the sample, making a total of 168 participants, due to possible dropout. Of the 168 questionnaires distributed, 166 were analyzed after excluding two incomplete surveys (response rate, 98.9%).

### Measurement

#### General and obstetric characteristics

All characteristics of the participants were measured using a self-reported questionnaire. Participants self-reported information on their age (year), gestational age (week), gravidity (number of pregnancies), parity (number of deliveries), and present job (occupied or not). Open-ended questions were used to elicit information on participants’ past and present health problems. The researchers determined and recorded participants’ trimester.

#### Anxiety

Anxiety was measured using the Korean version of the General Anxiety Disorder-7 (GAD-7) scale originally developed by Spitzer et al. [13]. The Korean version of the instrument was available on the Patient Health Questionnaire (PHQ) website (www.phqscreeners.com) and did not require permission to use. The tool consists of seven questions, and respondents are asked to answer the question, “Over the last 2 weeks, how often have you been bothered by the following problems?” Answers are given on a 4-point scale (0, not at all; 1, several days; 2, more than half of the days; and 3, nearly every day). Higher scores indicate higher anxiety. Cronbach’s alpha, which was used to determine the internal consistency, was .92 in the study by Spitzer et al. [13] and .87 in this study.

#### Depression

Depression was measured using the Korean version of the PHQ-2 developed by Spitzer et al. [14]. The Korean version of the instrument was available from the PHQ website (www.phqscreeners.com) and did not require permission to use. The tool consists of two questions for screening major depressive disorder in primary care: “During the last 4 weeks, how often have you been troubled by feeling down, depressed, or hopeless?” and “During the last 4 weeks, how often have you been troubled by little interest or pleasure in doing things?” Each question is answered on a 5-point scale, with 1 point indicating “not at all” and 5 points indicating “very much.” A higher score indicates a higher level of depression. Cronbach’s alpha was .73 in the study by Spitzer et al. [14] and .76 in this study.

#### Social support

To measure social support, we used the Perceived Social Support through Others Scale-8 (PSO-8) developed by Park [15] and shortened to eight items by Kim et al. [16] after receiving the approval of the original authors. The PSO-8 assesses three factors, with three questions on the quality of care provided, two questions on women’s personal attitudes, and three questions on the experience of stress during labor. It contains eight questions in total, and each question is answered on a 5-point Likert scale ranging from 1 point for “not at all” to 5 points for “very much.” Total possible scores range from 8 points to 40 points, with a higher score indicating a higher degree of social support. Cronbach’s alpha was .91 in the study by Kim et al. [16] and .95 in this study.

#### Physical health status

Physical health status was measured using the 1-item EuroQol visual analog scale (EuroQol VAS) developed by the European Quality of Life Group [17] and translated into Korean by the Korean Centers for Disease Control and Prevention. The tool was approved by the European Quality of Life Group. The EuroQol VAS consists of a single question answered on a self-reported basis asking the subjects to give a numerical health rating. In it, a 10-cm thermometer-like scale with graduations of 1 mm is depicted. At the bottom, 0 is labeled as the worst possible health rating, and at the top, 100 is labeled as the best possible health rating. A higher score indicates better perceived physical health according to the respondent.

#### Health-related quality of life

HRQoL was assessed using the 5-item physical health status survey (EuroQol 5-dimensions 3-levels, EQ-5D-3L) developed by the European Quality of Life Group [18] and translated into Korean by the Korean Centers for Disease Control and Prevention [19]. The tool was approved by the European Quality of Life Group. The EQ-5D-3L consists of five questions on mobility, self-care, usual activity, pain/discomfort, and anxiety/depression. Answers are given on a 3-point Likert scale, with a score of 1 indicating no problems (level 1), a score of 2 indicating some problems (level 2), and a score of 3 indicating extreme problems (level 3). A higher score indicates a lower HRQoL. In this study, HRQoL was analyzed based on the average score of the five questions. The single item of anxiety/depression captured the constructs differently from the GAD and PHQ regarding the symptom severity for a medical diagnosis. The EQ-5D-3L was designed to focus on symptom recovery, which is distinct from measuring depression and anxiety symptoms themselves [20]. The test-retest reliability of the original instrument as indicated by Cronbach’s alpha was .86 to .90, and the internal consistency reliability in this study as indicated by Cronbach’s alpha was .75.

### Data collection

We used convenience sampling to collect data from pregnant women who visited maternity centers between April 22 and May 2, 2023, at public health centers in Chuncheon, Gangwon Province, and Gongju, Chungcheongnam Province. The participants completed the surveys on a face-to-face basis in a maternal and child health center or classroom used for antenatal care education. Researchers and trained research assistants distributed recruitment notices and instructions related to the study to explain the purpose and content of the study. They then collected self-reported questionnaires from the participants, who provided informed consent. The questionnaire took 10 to 15 minutes to complete, and the participants filled them out at individual desks separated by at least 2 meters to avoid the disclosure of personal information. After completing the survey, participants were offered a gift worth 6,000 Korean won (approximately 5 US dollars).

### Data analysis

The collected data were analyzed using SPSS for Windows (version 26.0; IBM Corp., Armonk, NY, USA). The general and obstetric characteristics, anxiety, depression, social support, physical health status, and HRQoL of the participants were analyzed in terms of frequencies, percentages, means, and standard deviations. Differences in the degree of HRQoL were analyzed using the t-test and analysis of variance. Correlations between variables were analyzed using Pearson correlation coefficients. The factors that affected the participants’ HRQoL were analyzed using multiple regression analysis. The following assumptions for regression analysis were tested: the Shapiro-Wilks test for the normality of variables, the variance inflation factor for multicollinearity, and the Durbin-Watson value for the independence of residuals, equality of variance, and linearity.

## Results

### Participants’ general characteristics and differences in health-related quality of life based on their characteristics

The mean age of the participants was 34.39±4.29 years, and the mean gestational age was 24.37±8.11 weeks. A total of 56.0% of the participants were unemployed, and 78.9% of the participants had no past health problems. The vast majority of participants (94.0%) experienced no health problems in their current pregnancy. There were no significant differences in the HRQoL total mean score based on trimester (F=0.29, *p*=.746), gravidity (F=2.28, *p*=.105), parity (F=2.68, *p*=.071), job (t=1.27, *p*=.261), past health problems (t=–0.46, *p*=.640), and present health problems (t=–1.53, *p*=.127) (Table 1).

### Degree of anxiety, depression, social support, physical health status, and health-related quality of life

The participants had mean scores of 9.93±0.80 for anxiety, 2.80±0.88 for depression, 30.70±6.87 for social support, and 73.04±17.80 for physical health status. The mean score for HRQoL was 1.39±0.39, and the mean scores for the HRQoL subcategories were 0.32±0.56 for mobility, 1.10±0.35 for self-care, 1.32±0.55 for usual activity, 1.66±0.64 for pain/discomfort, and 1.55±0.62 for anxiety/depression (Table 2).

### Relationships among anxiety, depression, social support, physical health status, and health-related quality of life

The HRQoL of the participants showed statistically significant positive correlations to anxiety (r=.29, *p*=.001) and depression (r=.31, *p*<.001) and statistically significant negative correlations to social support (r=–.34, *p*<.001) and physical health status (r=–.44, *p*<.001) (Table 3).

### Impact of anxiety, depression, social support, and physical health status on health-related quality of life

The linear regression analysis assumptions were analyzed to determine the factors that affected the participants’ HRQoL. The diagnosis of collinearity, independence of residuals, normality, and linearity confirmed a Kolmogorov-Smirnov value of z=.105–.444, a Durbin-Watson value of 1.92, and a variance inflation factor of 1.063–2.147, and the slope of the P-P table was 45°; thus, the model was found to be appropriate. Physical health status (β=–.31, *p*<.001) and social support (β=–.21, *p*=.003) were the most important factors affecting the participants’ HRQoL, and the explanatory power of the model was 26.0% (F=15.50, *p*<.001) (Table 4).

## Discussion

This study found physical health status and social support to be the main factors affecting the HRQoL of pregnant women. This study adopted Spilker’s [8] quality of life model as its theoretical framework. This framework was partially supported since, among the mental, social, and physical domains, the social domain, which was measured in terms of social support, and the physical domain, which was measured in terms of physical health status, affected the HRQoL of pregnant women. This discussion, therefore, focused on the effects of physical health status and social support on HRQoL. Pregnancy is a normal part of life, but it is also a time during which women’s health is particularly vulnerable, and it involves major physical, mental, and social changes. This study is significant since it holistically identified the factors that most affect the HRQoL of pregnant women based on physical, mental, and social domains, mitigating the existing lack of research on the quality of life of pregnant women following the COVID-19 pandemic.

In this study, physical health status was the most significant factor affecting the HRQoL of pregnant women. Lau and Yin [21] also reported that lower physical health among pregnant women corresponded to a lower HRQoL. Among the common health problems experienced during pregnancy, nausea and back pain were the main symptoms associated with lower HRQoL [17]. A previous study found that pregnant women who participated in an aerobic exercise intervention had improved HRQoL in terms of physical function, pain, and general health domains compared to those who did not participate in the intervention [22]. However, significant differences in HRQoL were not observed among pregnant women who participated in another fitness intervention involving regular gym exercises compared to pregnant women who did not participate in the program [23]. According to a previous meta-analysis, moderately intense physical activity improves the quality of life of pregnant women [24]. Therefore, physical activity for pregnant women should be promoted. Monitoring physical fitness during pregnancy and providing tailored exercise interventions to pregnant women to prevent health problems will be a major factor in improving their HRQoL.

In this study, social support was the second major factor that affected the HRQoL of pregnant women. Previous studies have found the degree of social support to affect the HRQoL of pregnant women [24]. In addition, HRQoL tends to be lower among pregnant women with no spouse to provide social support [20]. A lack of emotional support from others can be perceived as rejection, exacerbating the psychological difficulties experienced by pregnant women in the wake of the COVID-19 pandemic. Globally, pregnant women have experienced high rates of depression, anxiety, and isolation during the COVID-19 pandemic, highlighting the importance of connecting with others socially and receiving their support [25]. Following the advent of the COVID-19 pandemic, interactive social support is urgently needed to ensure the mental health of pregnant women [26]. Social support is important in terms of both quantity and quality, and counseling from women’s health professionals can be an important source of social support [27]. Therefore, counseling and support from maternal and child health care professionals in addition to family members should be provided to pregnant women to improve their HRQoL.

In this study, anxiety and depression, as mental health indicators, were not found to be statistically significant factors affecting the HRQoL of pregnant women; however, they still showed a moderate correlation. Lau and Yin [21] also reported that worse mental health in pregnant women corresponded to a lower HRQoL. The mean score for anxiety among the pregnant women in this study was 9.93 points, with 4 points indicating mild anxiety, 10 to 14 points indicating moderate anxiety, and 15 to 21 points indicating severe anxiety [13]. The mean score for depression in this study as measured by the PHQ-2 was 2.80 points, indicating depression among the participants according to the instrument’s methodology, which classifies a positive response to any two items as an indicator of depression [13]. Social functioning, vitality, and emotional role had a moderate association with depression in pregnant women, and pain, physical health, physical functioning, and the physical role had a weak association [24]. A study of women in advanced countries found that pregnant women with a high level of depression had greater physical and social dysfunction, and nondepressed pregnant women had a better HRQoL than pregnant women with depression [10]. Prenatal depression was also associated with postpartum depression, suggesting that further efforts should be taken to improve the quality of life of pregnant women with a high level of depression given depression’s impact on pregnant women and their families [24].

The EQ-5D-3L is a widely used tool for assessing HRQoL, making it easy to compare scores across studies. In this study, the mean EQ-5D-3L score was 1.39, which was close to 1, indicating few problems in the respondents’ HRQoL. In studies of pregnant women by Camacho et al. [28] and Boutib et al. [24], the mean scores using the same instrument were 0.89±0.15 and 0.71±0.24, respectively, both of which were lower than the mean score in this study. A score range of 0.81-0.99 was reported in a 20-country wide-ranging survey of the general population [29]. The EuroQol VAS has been reported to range from 70.4 to 83.3 points. The EuroQol VAS score in this study was 73.04 points, which is similar. Therefore, the physical health status and HRQoL of pregnant women in this study were lower than those of women in general [30]. This finding is consistent with the finding that HRQoL is generally lower among pregnant women than among nonpregnant women and the population in general, especially in terms of the mental and physical domains of HRQoL [24].

In this study, we found that pregnant women, who are particularly vulnerable in the wake of the COVID-19 pandemic, experienced low social support, depression and anxiety, poor mental health, poor physical health status, and a decreased HRQoL. A longitudinal study of 12,007 pregnant women from 2020 to 2022 reported that increases in depression, anxiety, and stress coincided with timing of COVID-19 case surges [30]. Depression reportedly increased by 27.6% during the pandemic, while anxiety increased by 25.6%; younger people and women were more strongly affected, and less human mobility was associated with worse mental health [31]. Therefore, we also identified social support and physical health status as factors that affect the HRQoL of pregnant women. Therefore, to improve the HRQoL of pregnant women, maternal and fetal health professionals should strengthen their social support through counseling and implement interventions incorporating exercise and other activities to improve their physical health status.

This study has some limitations. First, it was conducted with pregnant women in the regions of Gangwon and Chungcheongnam Province only, and the participants were from rural areas rather than urban areas; therefore, caution should be exercised when generalizing the study results. Additionally, this study used self-reported surveys, which may have skewed the results since they did not reflect the objective health status of the women determined via direct measurement. In addition, due to the nature of the survey, which required the ability to read and write in Korean, it was impossible to include women of other ethnicities and races who may not have understood Korean.

Based on the findings of this study, we recommend conducting further surveys to identify the factors that influence the HRQoL of pregnant women across various regions. In particular, we suggest conducting a study to determine the factors that influence the HRQoL of pregnant women in different areas and compare the differences in anxiety, depression, social support, physical health status, and HRQoL between the prenatal and postnatal periods.

## Figures and Tables

**Table 1.** Characteristics of participants and differences of health-related quality of life (HRQoL) (N=166)

Variable	Categories	n (%)	Mean±SD (variable)	Range	Mean±SD (HRQoL)	t or F	*p*
Age (year)			34.39±4.29	23–46			
Gestational age (week)			24.37±8.11	3–38			
Trimester	First	14 (8.4)			1.37±0.39	0.29	.746
	Second	87 (52.4)			1.37±0.41		
	Third	65 (39.2)			1.41±0.39		
Gravidity	1	119 (71.7)		1–3	1.42±0.41	2.28	.105
	2	38 (22.9)			1.37±0.35		
	3	9 (5.4)			1.13±0.22		
Parity	0	121 (72.9)		0–2	1.41±0.40	2.68	.071
	1	35 (21.1)			1.39±0.35		
	2	10 (6.0)			1.12±0.21		
Job	Yes	73 (44.0)			1.40±0.38	1.27	.261
	No	93 (56.0)			1.38±0.40		
Past health problems^†^	Yes	35 (21.1)			1.42±0.45	–0.46	.640
	No	131 (78.9)			1.38±0.37		
Present health problems^‡^	Yes	10 (6.0)			1.58±0.58	–1.53	.127
	No	156 (94.0)			1.38±0.38		

† Cystitis, coronavirus disease 2019, hypothyroidism, Ménière disease, pyelonephritis, and thyroid cancer;

‡ Nausea, pruritis, diarrhea, constipation, hematuria, cough, hypothyroidism, and pelvic pain.

**Table 2.** Degree of anxiety, depression, social support, physical health status, and health-related quality of life (HRQoL) of participants (N=166)

Variable	Categories	Mean±SD	Range	Possible range
Anxiety		9.93±0.80	7–21	0–21
Depression		2.80±0.88	2–5	2–10
Social support		30.70±6.87	8–40	8–40
Physical health status		73.04±17.80	10–100	0–100
HRQoL	Mobility	1.32±0.56	1–3	1–3
	Self-care	1.10±0.35	1–3	1–3
	Usual activity	1.32±0.55	1–3	1–3
	Pain/Discomfort	1.66±0.64	1–3	1–3
	Anxiety/Depression	1.55±0.62	1–3	1–3
Total mean		1.39±0.39	1–3	1–3

**Table 3.** Relationships among age, anxiety, depression, social support, physical health status, and health-related quality of life (HRQoL) (N=166)

Factor	r (*p*)					
	Age	Anxiety	Depression	Social support	Physical health status	HRQoL
Age	1					
Anxiety	–.04 (.600)	1				
Depression	.01 (.940)	.58 (<.001)	1			
Social support	–.19 (.013)	–.20 (.008)	–.14 (.059)	1		
Physical health status	–.05 (.520)	–.33 (<.001)	–.29 (<.001)	–.29 (<.001)	1	
HRQoL	.12 (.098)	.29 (<.001)	.31 (<.001)	–.34 (<.001)	–.44 (<.001)	1

**Table 4.** Factors influencing participants’ health-related quality of life (N=166)

Factor	B	SE	ßβ	t	p
Anxiety	.008	0.01	.06	0.71	.474
Depression	.070	0.03	.15	1.88	.061
Social support	–.012	0.01	–.21	–3.06	.003
Physical health status	–.007	0.01	–.31	–4.19	<.001
Adjusted R^2^=26.0, df=4, F=15.50, *p*<.001					
